# Echocardiographic signs of subclinical cardiac function impairment in Duchenne dystrophy gene carriers

**DOI:** 10.1038/s41598-020-77882-6

**Published:** 2020-11-27

**Authors:** Vladimír Kincl, Roman Panovský, Martin Pešl, Jan Máchal, Lenka Juříková, Jana Haberlová, Lucia Masárová

**Affiliations:** 1 Department of Internal Medicine/Cardiology, Faculty of Medicine, St. Anne’s University Hospital, Masaryk University, Brno, Czech Republic; 2 International Clinical Research Center, St. Anne’s University Hospital, Brno, Czech Republic; 3 Department of Pathological Physiology, Faculty of Medicine, Masaryk University, Brno, Czech Republic; 4 Department of Pediatric Neurology, Faculty of Medicine, University Hospital Brno, Masaryk University, Brno, Czech Republic; 5 Department of Pediatric Neurology, Second Faculty of Medicine, University Hospital Motol, Charles University, Prague, Czech Republic; 6 Department of Biology, Faculty of Medicine, Masaryk University, Brno, Czech Republic

**Keywords:** Cardiomyopathies, Neuromuscular disease

## Abstract

To assess subclinical cardiac function impairment in Duchenne dystrophy (DMD) female carriers. Forty-four female subjects proved as DMD carriers underwent echocardiographic examination including tissue Doppler imaging (TDI) of mitral and tricuspid annulus. Seventeen age-matched healthy female subjects served as controls. A significant differences in peak systolic annular velocity (Sa) between carriers and controls were found for lateral and septal part of the mitral annulus and for tricuspid annulus (0.09 vs. 0.11 m/s, *p* < 0.001, 0.08 vs. 0.09 m/s, *p* < 0.01 and 0.13 vs. 0.14 m/s, *p* = 0.02 respectively). There was also difference in early diastolic velocity (Ea) of the septal part of the mitral annulus (0.11 vs. 0.13 m/s, *p* = 0.03). The subclinical deterioration of systolic function is presented even in asymptomatic DMD female carriers.

## Introduction

Duchenne (DMD) and Becker (BMD) muscular dystrophies are hereditary diseases linked on X chromosome. Thus, manifestation of skeletal muscle wasting, but also cardiomyopathy occurs in males, while female carriers of the defective *DMD* gene are perceived healthy. Nevertheless, they have only one functional variant of the gene on one of the X chromosomes.

In male patients, dystrophy has prevalence of 1/3500–6000^1^ affects primarily skeletal muscles, but also heart impairment may occur as a cardiomyopathy^2^. Cardiac involvement manifests as progressing decline in diastolic function, systolic ejection fraction, and fractional shortening^3^. Related is myocardial fibrosis, with muscle contraction impairment^4^.

There is number of proposed mechanisms of the disease etiology, primary sarcolemmal tears^5^ as consequence of non-functional dystroglycan complex has number of consequences, e.g. increase oxidative stress, ion channel disturbances as well as numerous molecular pathways alteration^6–8^ eventually leading to impaired heart muscle regeneration, possibly due to stem cell depletion^9^ The severity of cardiomyopathy is not always in correlation with skeletal myopathy and cardiac impairment occurs long before clinical symptoms^10^. In female carriers, the clinical symptoms are mostly not presented, thus cardiac function has not been studied in depth. Nevertheless, in case studies were published severe heart failure episodes in different settings as peripartum cardiomyopathy, perioperative stress and others^11–15^, possibly leading even to heart transplantation^16^. The muscular symptoms of DMD carriers may vary from asymptomatic individuals to muscular weakness, frequent falling, difficulties in running or jumping or climbing stairs ^17^. Still complex prospective randomized studies are missing.

In our previously published study^4^ in young males with manifest Duchenne dystrophy we used cardiac magnetic resonance (CMR) to assess the cardiac function and early signs of affection of the heart by T1 mapping because echocardiography has some difficulties due to skeletal deformities and narrow intercostal spaces. Female carriers do not present rib cage anomalies, thus we used echocardiography with tissue Doppler imaging as first line method to assess subclinical cardiac dysfunction. Our another study^18^ with asymptomatic DMD female carriers with preserved left ventricular (LV) ejection fraction (EF) proved lower global longitudinal strain, global circumferential strain and radial strain on CMR in comparison with healthy controls. The subjects enrolled in this study had no history of weakness, falling or problems with stairs climbing or walking longer distances (more than 1 km), as well as no history of heart disease. They also had absence of cardiac symptoms like severe dyspnea, ankle swelling or palpitations. The aim of this study is to assess detectable changes of tissue Doppler parameters in asymptomatic carriers in comparison with healthy control subjects.

## Patients and methods

Forty-four female subjects with genetically diagnosed presence of DMD allele underwent echocardiography examination on standard ultrasound device Vivid 9 (GE Healthcare, Wisconsin, USA). Measurements of heart dimensions, LVEF, valvular morphology and parameters and tissue Doppler imaging of mitral and tricuspid annular velocities were performed. The dimensions were measured from parasternal long axis view, EF was calculated according to Simpson’s rule. Tissue Doppler curves were obtained from standard apical four-chamber view, the peak systolic (s´), early diastolic (e´) and late diastolic (a´) velocities of lateral and septal part of the mitral annulus and lateral tricuspid annulus were obtained. The ratio between early diastolic (E) wave of transmitral flow and average of mitral annular lateral and septal velocities (E/e´) was calculated^19^. The demographic and clinical characteristics of cohort are presented in Table 1. The echocardiographic parameters were compared to control group of 17 healthy female subjects, without known or echocardiographically detectable heart disease, with mean age of 36 years.

**Table 1:** Characteristics of Duchenne dystrophy carriers cohort.

N	44
Age, years (mean ± SD)	38.8 ± 10.3
Body mass index (mean ± SD)	23.3 ± 4
Heart disease, N ( %)	0 (0%)
Hypertension, N ( %)	2 (5%)
Diabetes mellitus, N ( %)	2 (5%)
Hyperlipoproteinaemia, N ( %)	5 (11%)

*SD* standard deviation.

### Ethics approval and consent to participate

The study was performed in accordance with the Declaration of Helsinki (2000) of the World Medical Association, and was approved by the institutional ethics committee (University Hospital Brno, reference number 20130410-03). Written informed consent was obtained from the subjects and/or their legally authorized representative.

## Statistical analysis

The female carriers of dystrophin loss-of-function mutation were statistically compared with the age-matched female controls. Because most variables were either integers or did not follow Gaussian distribution, non-parametric Mann–Whitney U-test was used for the statistical comparison. The value of α = 0.05 was set as the significance level in all tests. All analyses were performed using Statistica software (version 13.3, Tibco software, USA).

## Results

**Figure 1:**
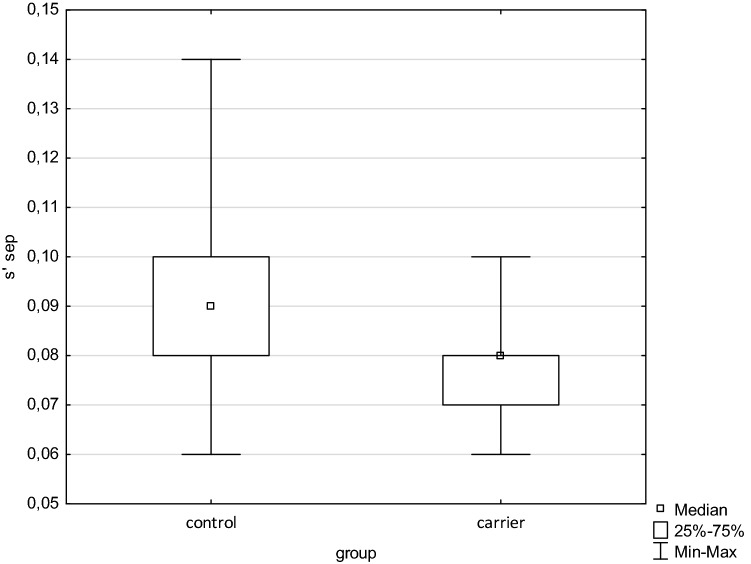
Box and whisker plot of s´ from septal part of the mitral annulus. Significant difference between groups *p* < 0.01 (s´—tissue Doppler peak systolic annular velocity).

**Figure 2:**
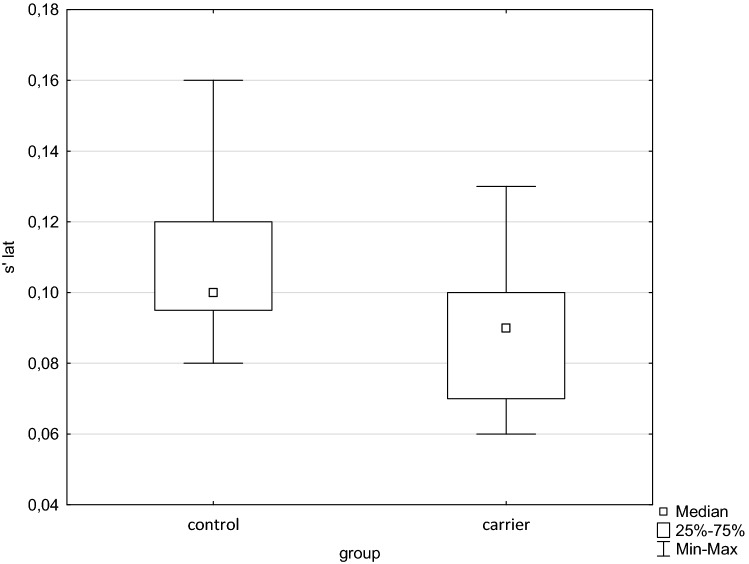
Box and whisker plot of s´ from lateral part of the mitral annulus. Significant difference between groups *p* < 0.001 (s´—tissue Doppler peak systolic annular velocity).

**Figure 3:**
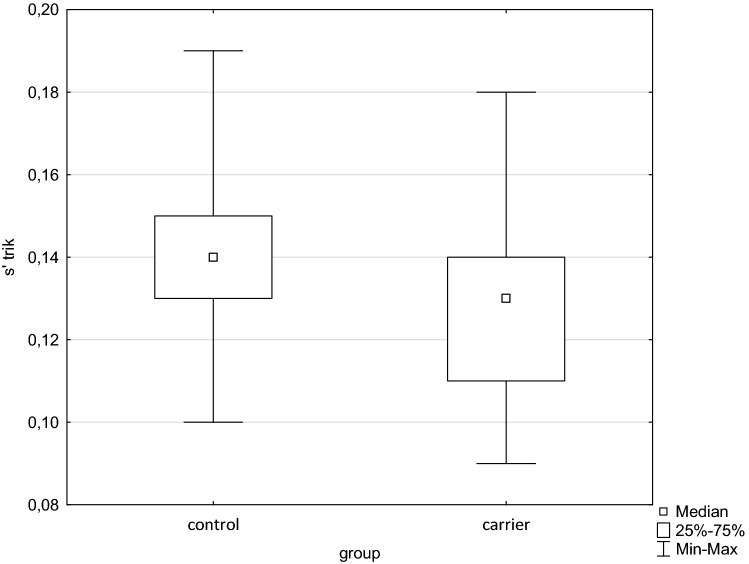
Box and whisker plot of s´ from tricuspid annulus. Significant difference between groups *p* = 0.02 (s´—tissue Doppler peak systolic annular velocity).

The both groups did not differ in age, left and right ventricular end-diastolic diameter, interventricular septal, posterior wall and left atrial diameter, and ejection fraction. There was only slight difference in LV end-systolic dimension (carriers vs. controls, 29 vs. 27 mm, *p* = 0.01). No serious valvular disease was found in carriers group. The LV diastolic parameters (E/A and E/e´ ratio) were also without significant difference. However, in tissue Doppler parameters were differences in mitral e´ wave from septal part of mitral annulus, a´ wave from septal and lateral mitral annulus, and s´ wave from both parts of mitral and also tricuspid annulus. All above mentioned annular velocities were significantly lower in carriers in comparison with controls. The basic echocardiographic data are presented in Table 2, tissue Doppler parameters in Table 3, box and whisker plots of Sa waves are shown on Figs. 1, 2 and 3.

**Table 2:** Basic heart dimensions, ejection fraction and diastolic parameters.

	Carriers (N = 44)	Controls (N = 17 )	*p*
DD (mm)	43 ± 4	42 ± 4	NS
DS (mm)	29 ± 4	27 ± 3	0.01
LA ( mm)	34 ± 4	34 ± 4	NS
I vs. (mm)	9 ± 1	9 ± 1	NS
PW (mm)	9 ± 1	9 ± 1	NS
RV (mm)	24 ± 4	25 ± 3	NS
LV EF (%)	64 ± 4	65 ± 4	NS
E (m/s)	0.81 ± 0.13	0.85 ± 0.18	NS
A (m/s)	0.53 ± 0.11	0.6 ± 0.1	NS
E/A	1.59 ± 0.37	1.42 ± 0.35	NS
e’ (m/s)	0.13 ± 0.03	0.13 ± 0.03	NS
E/e´	6.6 ± 1.3	6.1 ± 1.2	NS

Data are presented as mean ± SD.

DD—left ventricular end-diastolic diameter, DS—left ventricular end-systolic diameter, LA—left atrium diameter, IVS—interventricular septum diameter, PW—left ventricular posterior wall diameter, RV—right ventricular end-diastolic diameter, LV EF—left ventricular ejection fraction, E—transmitral early diastolic wave, A—transmitral late diastolic wave, E/A—ratio between E and A wave, E/e´—ratio between E wave and mean value of e´ tissue Doppler wave from lateral and septal mitral anulus, e´—mean value of e´ tissue Doppler wave from lateral and septal mitral anulus.

**Table 3:** Tissue Doppler parameters.

	Carriers (N = 44)	Controls (N = 17 )	*p*
e´sept (m/s)	0.11 ± 0.02	0.13 ± 0.02	0.03
a´ sept (m/s)	0.09 ± 0.02	0.10 ± 0.02	0.05
s´ sept (m/s)	0.08 ± 0.01	0.09 ± 0.01	< 0.01
e´ lat (m/s)	0.14 ± 0.03	0.15 ± 0.03	NS
a´ lat (m/s)	0.08 ± 0.02	0.09 ± 0.02	< 0.01
s´ lat (m/s)	0.09 ± 0.02	0.11 ± 0.02	< 0.001
e´avg (m/s)	0.13 ± 0.03	0.13 ± 0.03	NS
e´ tric (m/s)	0.15 ± 0.07	0.16 ± 0.03	NS
a´ tric (m/s)	0.13 ± 0.04	0.11 ± 0.03	NS
s´ tric (m/s)	0.13 ± 0.02	0.14 ± 0.02	0.02

e´—peak early diastolic velocity, a´—peak late diastolic velocity, s´—peak systolic velocity, sept—septal part of the mitral annulus, lat—lateral part of the mitral annulus, avg—average from the septal and lateral part of the mitral annulus, tric—tricuspid annulus.

## Discussion

The main result of our study was the fact that even asymptomatic DMD carriers without significant systolic dysfunction have signs of subclinical systolic function impairment. Although strain and speckle tracking has become mostly used techniques for detail assessment of cardiac function by echocardiography, tissue Doppler imaging (TDI) remains one of the most powerful and well proved method in heart dysfunction diagnostics^19,20^. The mitral annulus peak velocities are valuable indicators of long-axis left ventricular motion and thus of LV systolic and diastolic function^21^. The peak systolic velocity (s´) is very sensitive marker of LV dysfunction, even in subjects with preserved ejection fraction (EF) or in diabetic patients without cardiac disease^22^. Reduced TDI velocities were also found in asymptomatic carriers of hypertrophic cardiomyopathy mutations without presence of cardiac hypertrophy^23^. The early diastolic annular velocity is one of the most powerful predictive echocardiographic parameters^24,25^. The e´ parameter is very sensitive marker of impaired diastolic function and it decreases in all stages of diastolic dysfunction^19^. In advanced phases of diastolic failure, the e´ velocity remains low, but the E wave velocity is high as LV filling pressure increases, so the E/e´ ratio increases as well^26,27^. Although TDI values obtained from Duchenne dystrophy carriers in our study were in normal range according to age^28^, significant difference particularly in systolic parameters (s´) was found in comparison with age-matched control group. Several subjects had s´ from lateral mitral annulus below 5th percentile of normal values range. On the other hand, diastolic parameters did not differ so clearly, only septal e´ and Aa and lateral a´ were substantially lower. The E/e´ ratio was normal in both groups without significant difference. The prevalence of cardiomyopathy in female DMD carriers varies in wide range and does not correlate with phenotype, muscle symptoms, creatinine kinase levels or age^13,15,29^. Also ECG changes, including Q-waves in the lateral leads (I, AVL, V6), R- mm in V1 and R/S in V2 were described in DMD carriers ^30^. In our study were enrolled only asymptomatic carriers without developed cardiomyopathy, but the slight impairment of systolic LV and RV parameters is presented even with normal diastolic function. This is in concordance with previous studies^31^;, where impairment of systolic function was more pronounced than in diastolic echocardiographic parameters. However, this study assessed heart dimensions and fractional shortening, not tissue Doppler parameters. Also prevalence of dilated cardiomyopathy was relatively high (8.2%) in contrast with our study, which comprises asymptomatic subjects with normal ejection fraction and without significant LV dilation. So there is a premise, that DMD even at early stages affects primarily contractile function of cardiomyocytes, without influence on relaxing process and LV filling patterns. There were some limitations in our study. First, small number of patients, but on the other hand, DMD is a rare disease so it is difficult to enroll more subjects in nationwide study within the Czech Republic. The another one is absence of use of strain in echocardiography, but in the case of diffuse myocardial impairment tissue Doppler echocardiography provides sufficient information of heart dysfunction and it is easier and faster to obtain.

## Conclusion

The subclinical deterioration of systolic function is present even in asymptomatic DMD female carriers. Tissue Doppler echocardiography is very appropriate, fast, simple and non-invasive method to assess subclinical cardiac dysfunction in DMD carriers. Larger studies with follow-up of subjects are needed.

## Data Availability

The datasets analyzed during the current study are available from the corresponding author upon reasonable request.
